# Surgery

**Published:** 1904-08-13

**Authors:** 


### Progress in Medicine and Surgery.

### SURGERY.

Aseptic Surgery in Theory and Practice is the subject dealt with by Lockwood [1] in the Lettsomian lectures. The experiences and conclusions of one who has made so special a study of this subject are most valuable and interesting. The division of surgeons into two schools—the aseptic and the antiseptic—is most undesirable. All modern surgery is aseptic, in that the end to be attained is asepsis. The means to attain that end vary in detail. Some may make more use of chemical antiseptics than others, but are just as much entitled to call themselves aseptic surgeons as are those who claim to dispense with antiseptics. So far no means have been devised of rendering the surgeon's hands, the patient's skin, or the wound approximately sterile without the aid of chemical disinfectants. The author's principle is to sterilise by heat whatever can be so sterilised, and for the rest to use chemical antiseptics. The air must be a source of infection. Every open wound is to a greater or less degree infected in direct ratio to the length of exposure and amount of manipulation. It seems irrational to make no attempt to get rid of the bacteria which must gain entrance to every wound, when chemical antiseptics can kill, or at any rate attenuate, them without in any way damaging the tissues, or interfering with the healing process. For this purpose he finds a solution of 1 in 4000 biniodide of mercury most satisfactory. Theoretical perfection is hard to obtain, but the effort to obtain it is useful, and will lead to improved results. A hand, if not perfectly sterile, when soaked with an antiseptic solution is less likely to infect a wound than a hand which is not. He is sceptical as to the possibility of disinfecting a once infected wound; chemical antiseptics are working at a great disadvantage owing to the coagulation of exudates, the infection from the atmosphere is mostly superficial, and so, perhaps, can be dealt with by an antiseptic lotion from time to time during an operation, before the bacteria become rubbed into the wound by manipulation. What may be an excellent antiseptic in the laboratory may act but feebly in a wound, and *vice versâ*; for example, iodoform has a very poor reputation in the bacteriological laboratory, yet clinically no one can deny its value in foul and septic wounds. The organisation of an operation does not receive the attention it should. In an aseptic operation every detail must be perfect, and every one engaged in it consistent. He thinks it probable that the system of assistants will require modification. It is most important for the surgeon to be assisted by one who is not only thoroughly educated and practised in aseptic details, but who is also conversant with the operator's methods. House surgeons and dressers do their work admirably and with great devotion, but as soon as they are becoming really efficient their term of office expires. He pleads for simplicity and uniformity as great preventives of chance infection, the elimination of each unnecessary pair of hands lessens the danger of infection. As far as possible the surgeon's hands alone should be put into the wound, or handle the ligatures, instruments, or dressings. A very important point to which he calls attention, and one which seems to be but little recognised, is the danger of talking during an operation, this may be a fertile source of wound infection from the passage of particles of saliva from the mouth, such particles have been shown to be constantly ejected from the mouth during conversation. Discussing gloves for operating, he considers cotton gloves to be absolutely useless as a means of protecting the wound, if there be infection beneath it can easily soak through when the glove is wet. Rubber gloves are easily sterilised, but are generally torn or punctured; they very seriously impair the sense of touch, and he finds them thus useless to him in abdominal work; they are useful in dealing with septic cases, and should be always used to protect the surgeon's hand, and so avoid the risk of the surgeon infecting a subsequent operation, since it takes a considerable time to disinfect the hands after manipulating septic material. For buried ligature or suture he favours silk; he considers that the boldness with which a surgeon buries silk is a good test of his proficiency in asepsis. Absorbable ligatures are troublesome to sterilise, and may be absorbed too soon; they should be used in septic cases. Silk is easily sterilised by boiling for half an hour; it should be used fresh from the steriliser, as silk that is used out of strong carbolic solutions irritates the tissues. Each needle-hole may be observed to have a ring of inflammation around it when used in the skin and there is probably a similar effect in the deeper tissues. Silk ligatures, especially in muscle, should not be tied so tightly as to strangulate tissue, neglect of this precaution he believes to be the cause of extrusion of the sutures after primary union of the wound has been obtained; the healthy tissues can deal with many of the bacteria that gain entrance at the operation, but in strangulated and dead tissue the organisms will flourish and infect the ligature. Marine sponges are undoubtedly superior to cotton swabs if they can be sterilised; he believes that they are sterile prepared by the sulphurous acid method; once a sponge has touched anything infective it must be destroyed. Sepsis arising in clean wounds is of two principal varieties—progressive suppuration with general infection, and slight local suppuration, often due to extravasation of blood, or to necrosis of an edge of skin, he has not had a case of the former variety or acute sepsis during a period of three years in which he has tabulated his results. In tabulating cases as to healing results another class besides septic and aseptic wounds is required; he proposes placing these cases in a class labelled "secondary skin infection following necrosis of skin." Accurate apposition of the skin is of great importance; when the apposition is good the skin soon unites and has formed an effectual barrier to infection before any bacteria which may have survived in sweat glands or hair follicles have had time to multiply and invade the deeper tissues. Drainage tubes are a source of danger in clean wounds, forming a tract along which bacteria can travel; the use of drainage tubes has now, however, fallen very much into abeyance. The essential points of a dressing, in his opinion, are sterility and dryness; next to the skin he places a strip of silver foil, which is supposed to have a retarding influence on bacterial growth, while it excludes air, and it separates an antiseptic dressing, if such be used, from the wound. For a dressing he prefers plain sterile gauze and absorbent wool.

[1] Brit. Med. Jour., Feb. 6, 13, 20, 1904.

(*To be continued.*)

